# Oral Health Considerations for Adults Aged 18 Years or More Seeking Dental Care in the Past Year: A CDC Oral Health Data Analysis

**DOI:** 10.7759/cureus.52200

**Published:** 2024-01-13

**Authors:** Emeka Okobi, Okelue E Okobi, Ademiluyi B David, Victor C Ofochukwu

**Affiliations:** 1 Dentistry, Ahmadu Bello University Teaching Hospital Zaria, Abuja, NGA; 2 Family Medicine, Larkin Community Hospital Palm Springs Campus, Miami, USA; 3 Family Medicine, Medficient Health Systems, Laurel, Maryland, USA; 4 Family Medicine, Lakeside Medical Center, Belle Glade, USA; 5 Medical Laboratory Sciences, Asokoro General Hospital Abuja, Abuja, NGA; 6 Medicine, Ebonyi State University, Abakaliki, NGA; 7 Medicine and Surgery, Hospital Corporation of America (HCA) Hospital Pearland, Pearland, USA

**Keywords:** socioeconomic disparities, dental visit, adults aged 18+, complete tooth loss, oral health

## Abstract

Background: Oral health is an essential aspect of overall well-being, with regular dental care being fundamental to its maintenance. This study focuses on understanding dental care utilization among adults aged 18 and above who have visited a dentist or dental clinic in the past year, aiming to uncover patterns, disparities, and determinants of oral health practices within this demographic.

Methods: Data from the U.S. Centers for Disease Control and Prevention (CDC) Oral Health dataset were utilized to conduct this analysis. The dataset encompasses a diverse and nationally representative sample of adults aged 18 and above. The study explored the proportion of adults who sought dental care between 2008 and 2020, further stratified by demographic variables including age, gender, income, education, and race. The analysis provides insights into the prevalence of dental care utilization and the role of demographic factors in shaping oral health behaviors.

Results: The study found that 64.8% (n =397,291; 95% CI: 64.4 - 65.2) of adults aged 18 and above visited a dentist or dental clinic in 2020. Subgroup analysis revealed variations in dental care use by age, gender, income, education, and race. Among genders, 67.4% (n = 150,510; 95% CI: 66.9 - 67.9) of females sought care in 2020, compared to 61.9% (n = 116,535; 95% CI: 61.4 - 62.4) of males. Those earning >$50,000 had the highest proportion, 75.3% (n = 13,363; 95% CI: 74.8 - 75.8), seeking care. Among racial groups, White adults had the highest proportion, 68.4% (n = 204,486; 95% CI: 68.0 - 68.8) in 2020. In education groups, college graduates or professionals had the highest, 77.3% (n = 121,800; 95% CI: 76.8 - 77.8) in 2020. Among ages, adults aged 65+ had the highest proportion, 67.1% (n = 96,012; 95% CI: 66.4 - 67.8) in 2020. However, as age decreased, dental visit proportion generally remained within the same range.

Conclusion: This study enhances our understanding of dental care utilization patterns within the studied population, shedding light on disparities in oral health practices. Moreover, it provides insight into how demographic factors shape dental/oral healthcare-seeking behaviors. Ultimately, these insights guide efforts to improve oral health outcomes and well-being within this population.

## Introduction

Oral health is a fundamental aspect of overall health and well-being, with a profound impact on an individual's quality of life. Regular dental visits play a crucial role in maintaining oral hygiene and preventing potential oral health complications [1]. Globally, dental caries remain highly prevalent among adults aged 18 years and above, with 35% of the cases remaining untreated. In the United States, approximately 90% of adults aged 20-64 have experienced dental caries [2-3]. Periodontal disease, including gingivitis and periodontitis, affects a substantial number of adults. About 47% of adults aged 30+ in the United States have some form of periodontal disease, with variations based on location and socioeconomic factors [4]. Tooth loss is significant, often tied to untreated dental caries and periodontal disease. A study by Urzua et al. reported that adults exhibit an average of 5 to 10 missing teeth attributed to dental caries. By the age of 65, the average United States adult has lost around six or more teeth due to tooth decay and dental issues [5-6]. 

Oral health considerations for adults who have visited a dentist involve understanding the pathophysiology of plaque formation, cavities, gum disease, and the importance of preventive measures. These diseases can significantly impact an individual's oral functionality, aesthetics, and overall health. The decision to seek dental care reflects a proactive stance towards maintaining oral health, enabling the identification and management of oral health diseases at an early stage. Regular dental visits not only facilitate professional care but also contribute to early detection and personalized guidance for maintaining optimal oral health, which in turn can impact overall well-being [1,7-8]. By focusing on the adult age group, we can gain insights into the prevalence of dental care visits, potential barriers faced by certain subgroups, and the impact of socio-economic and cultural factors on oral health behaviors [9].

This study aims to examine the dental care-seeking behavior of adults who visit a dentist or dental clinic. Additionally, the objective of this study is to understand the utilization of dental care in adults aged 18 years and above and to disclose the patterns, disparities, and key determinants of oral health practices within the age group. The analysis draws upon data extracted from the Centers for Disease Control and Prevention (CDC) Oral Health dataset, encompassing a comprehensive collection of oral health-related information from a wide demographic spectrum [10]. By delving into this data, we can uncover patterns and variations in dental care utilization, leading to a more nuanced understanding of how individuals in different demographic segments prioritize and engage with their oral health needs. Understanding the dynamics of dental care utilization among adults aged 18 and above is essential for designing targeted interventions, promoting oral health education, and improving access to dental care services. Identifying disparities and trends in dental care-seeking behavior can inform public health policies, clinical practices, and community outreach efforts aimed at enhancing oral health outcomes for this diverse and vital segment of the population [9-11].

## Materials and methods

Study design

This research employed a retrospective data analysis methodology to investigate oral health considerations among adults who visited a dentist or dental clinic within the past year. The study leveraged the extensive dataset provided by the CDC, encompassing a broad range of demographic variables. The analyses for this study were conducted between August 7, 2023, and August 16, 2023, within the specified timeframe.

Data source and data collection

The primary data source for this analysis was the CDC's Oral Health Surveillance System, a comprehensive repository containing survey data collected through national oral health surveys, health examinations, and health records. The dataset spanned between 2012 and 2020, enabling a longitudinal assessment of oral health trends and considerations. Through initiatives such as the National Health and Nutrition Examination Survey (NHANES), the Behavioral Risk Factor Surveillance System (BRFSS), and the National Survey of Children's Health (NSCH), the CDC systematically gathered information on various aspects of oral health, including dental caries, gum diseases, dental visits, hygiene practices, and access to care [10-11]. The CDC's Oral Health Surveillance System collected data through various methods, including standardized questionnaires, dental examinations, and clinical assessments conducted by trained oral health professionals. Demographic information such as age, gender, race, income, and education level were captured alongside oral health data, including dental visits, oral hygiene practices, dental conditions, and treatment history.

Inclusion criteria

To ensure relevance and accuracy, the study included adults aged 18 and above who had visited a dentist or dental clinic within the past year. Individuals meeting this criterion were considered actively engaged in oral health care and were the focus of this analysis.

Data analysis

During the data analysis phase, we summarized aggregate data spanning 2012 to 2020, focusing on relevant patient characteristics. Descriptive statistical analysis was conducted to ascertain the percentage of adults who visited dental clinics. The proportions were derived by dividing the number of individuals with complete tooth loss in each demographic category by the total count within that category. We calculated frequencies, percentages, and 95% confidence intervals (CI) for variables including age, gender, income, and education level.

Ethical considerations

This study adhered to ethical guidelines and ensured the protection of participant privacy and confidentiality. As the dataset was de-identified and publicly accessible, no additional ethical approvals were required for this secondary data analysis.

## Results

The analysis of dental care utilization among adults who visited a dentist provided crucial insights into the prevalence of seeking dental care services within this population. The findings presented were based on the analysis of data extracted from the CDC Oral Health dataset, offering an overview of dental care-seeking behavior across various demographic segments. The results revealed the overall proportion of adults aged 18 and above who had visited a dentist or dental clinic in the past year. In 2020, 64.8% (n = 397,291; 95% CI: 64.4 - 65.2) of adults within this age range reported visiting a dentist or dental clinic in the past year.

The proportion of adults seeking dental care by gender

**Figure 1:**
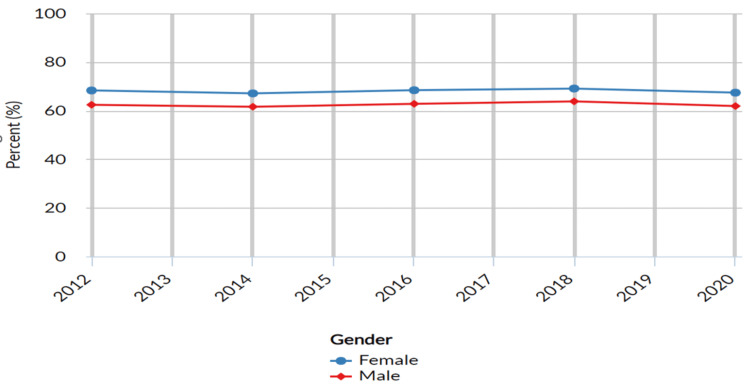
Classification by gender of individuals who have visited a dentist or dental clinic in the past year United States- All available years, Adults aged 18 years+ who have visited a dentist or dental clinic in the past year, Breakdown – Gender, Response-Yes

The gender categories used in the analysis were Male and Female. These categories were employed to explore variations in dental care utilization between individuals of different genders. The results indicated variations in the proportion of adults aged 18 years and above who had visited a dentist or dental clinic in the past year based on gender (See Table 1 below). Of the patients who sought dental care in 2021 in comparison to 2019, the proportion of females seeking dental care in 2020 was higher, at 67.4% (n = 150,510; 95% CI: 66.9 - 67.9), compared to males seeking dental care, at 61.9% (n = 116,535; 95% CI: 61.4 - 62.4) (See Figure 1 below).

**Table 1:** Total adults and gender-wise classification who have visited a dentist or dental clinic in the past year CI: Confidence interval, (-): Intentionally left blank, n: number

-	Yes	No	Female	Male
2020				
Percent (%)	64.8	35.5	67.4	61.9
CI	64.4 - 65.2	35.1 - 35.9	66.9 - 67.9	61.4 - 62.4
n	397291	397291	150510	116535
2018				
Percent (%)	66.5	33.7	69.1	63.8
CI	66.2 - 66.8	33.4 - 34.0	68.7 - 69.5	63.4 - 64.2
n	432676	432676	167152	128238
2016				
Percent (%)	65.7	34.3	68.4	62.8
CI	65.4 - 66.0	34.0 - 34.6	68.0 - 68.8	62.4 - 63.2
n	323299	149527	188583	134687
2014				
Percent (%)	64.4	35.6	67.1	61.6
CI	64.1 - 64.7	35.3 - 35.9	66.7 - 67.5	61.2 - 62.0
n	307508	144386	184153	123355
2012				
Percent (%)	65.4	34.6	68.3	62.4
CI	65.1 - 65.7	34.3 - 34.9	67.9 - 68.7	61.9 - 62.9
n	314992	148066	192566	122426

Adults who have visited a dentist or dental clinic in the past year based on income

The income categories were divided into five types: less than $15,000; $15,000 to $24,999; $25,000 to $34,999; $35,000 to $49,999; and more than $50,000. These categories were utilized to examine variations in dental care utilization among individuals with differing income levels. The results revealed variations in the proportion of adults who visited a dentist or dental clinic in the past year based on different income categories (Table 2 below).

**Table 2:** Classification by income category of individuals who have visited a dentist or dental clinic in the past year CI: Confidence interval, (-): Intentionally left blank, n: number

-	Less than $15,000	$15,000 - $24,999	$25,000 - $34,999	$35,000 - $49,999	$50,000+
2020					
Percent (%)	44.8	49.5	55.2	61.3	75.3
CI	43.2 - 46.4	48.4 - 50.6	53.8 - 56.6	60.2 - 62.4	74.8 - 75.8
n	11178	24004	17989	28338	133633
2018					
Percent (%)	46.8	51.2	57.3	64.3	78.1
CI	45.6 - 48.0	50.3 - 52.1	56.2 - 58.4	63.3 - 65.3	77.7 - 78.5
n	15122	29243	22484	33240	146137
2016					
Percent (%)	44.4	50.5	56.6	64.7	78.3
CI	43.4 - 45.4	49.7 - 51.3	55.6 - 57.6	63.8 - 65.6	77.9 - 78.7
n	16488	33669	25792	39224	155330
2014					
Percent (%)	42.8	47.8	57.7	64.7	78.8
CI	41.8 - 43.8	47.0 - 48.6	56.7 - 58.7	63.9 - 65.5	78.4 - 79.2
n	16903	33148	26416	39202	145886
2012					
Percent (%)	43.2	49.2	58.2	67.2	80.3
CI	42.2 - 44.2	48.4 - 50.0	57.2 - 59.2	66.4 - 68.0	79.9 - 80.7
n	20357	36759	28634	41754	144618

**Figure 2:**
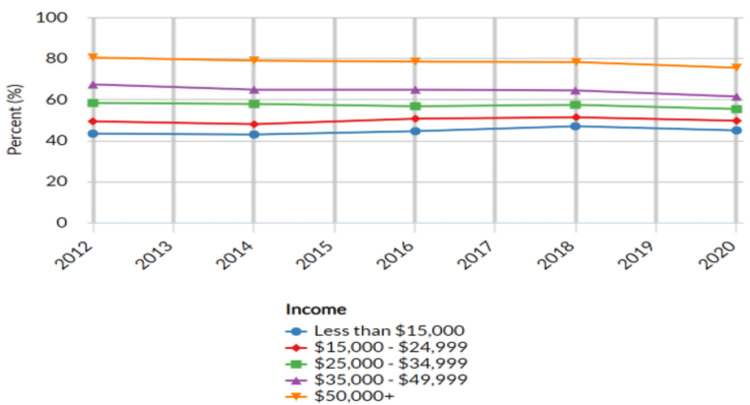
Adults who have visited a dentist or dental clinic in the past year based on income United States- All available years, Adults aged 18 years+ who have visited a dentist or dental clinic in the past year, Breakdown – Income, Response-Yes

Among the income groups considered, the highest proportion of adults seeking dental care in the past year was observed among those with incomes exceeding $50,000, reporting a proportion of 75.3% (n = 13,363; 95% CI: 74.8 - 75.8) in 2020. The proportion decreased gradually as income levels decreased. For individuals with incomes between $35,000 and $49,999, the proportion was 61.3% (n = 28,238; 95% CI: 60.2 - 62.4). Similarly, among those with incomes between $25,000 and $34,999, 55.2% (n = 17,989; 95% CI: 53.8 - 56.6) sought dental care. In the $15,000 to $24,999 income range, the proportion was 49.5% (n = 24,004; 95% CI: 48.4 - 50.6), while among those with incomes less than $15,000, the proportion seeking dental care was 48.4% (n = 11,178; 95% CI: 43.2 - 46.4) (Figure 2 below).

Adults who have visited a dentist or dental clinic in the past year based on race

Within the study population, the proportion of adults who visited a dentist or dental clinic in the past year varied across different racial groups. The results indicated variations in the proportion of adults who had visited a dentist or dental clinic in the past year across different racial categories (Table 3 below).

**Table 3:** Classification by race for individuals who have visited a dentist or dental clinic in the past year CI: Confidence interval, (-): Intentionally left blank, n: number

-	White	Black	Hispanic	Other	Multiracial
2020					
Percent (%)	68.4	60.1	56.4	63.7	59.9
CI	68.0 - 68.8	59.0 - 61.2	55.2 - 57.6	61.8 - 65.6	57.5 - 62.3
n	204486	17431	20699	13873	4908
2018					
Percent (%)	69.7	60.5	59.1	67.7	61.3
CI	69.4 - 70.0	59.5 - 61.5	58.1 - 60.1	66.2 - 69.2	59.3 - 63.3
n	228248	20793	22204	14382	5028
2016					
Percent (%)	69	59.6	56.4	68.7	59.7
CI	68.7 - 69.3	58.7 - 60.5	55.4 - 57.4	67.3 - 70.1	57.4 - 62.0
n	257486	22646	19174	13051	5446
2014					
Percent (%)	68.4	56.5	54.2	65.8	59.2
CI	68.1 - 68.7	55.6 - 57.4	53.2 - 55.2	64.2 - 67.4	57.1 - 61.3
n	249342	19673	16787	11713	5019
2012					
Percent (%)	69.2	57.5	55.2	67.7	59.8
CI	68.9 - 69.5	56.5 - 58.5	54.1 - 56.3	66.2 - 69.2	57.5 - 62.1
n	254149	22323	16847	12751	5078

**Figure 3:**
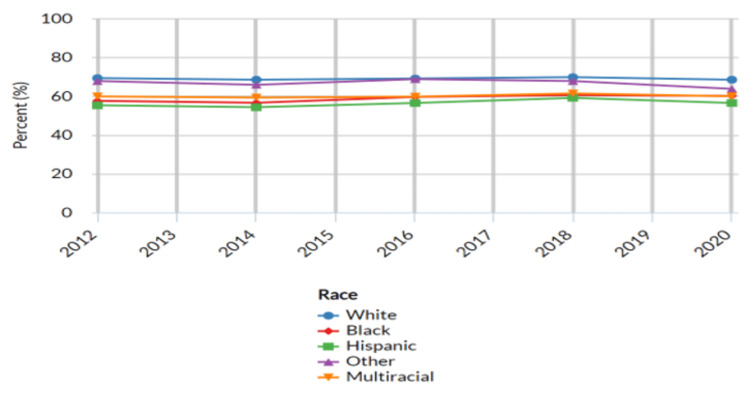
Adults who have visited a dentist or dental clinic in the past year based on race United States- All available years, Adults aged 18 years+ who have visited a dentist or dental clinic in the past year, Breakdown – race, Response-Yes

Among the racial groups considered, the highest proportion of adults who sought dental care in the past year was observed among White adults, with a reported proportion of 68.4% (n = 204,486; 95% CI: 68.0-68.8) in the year 2020. This was followed by Black adults with 60.1% (n = 17,431; 95% CI: 59.0-61.2) and multiracial adults with 59.9% (n = 4,908; 95% CI: 57.5-62.3) seeking dental care in the same period. Hispanic adults reported a proportion of 56.4% (n = 20,699; 95% CI: 55.2-57.6) who had visited a dentist or dental clinic in the past year. The "Other" racial category, comprising individuals from various racial backgrounds, exhibited a proportion of 63.7% (n = 13,873; 95% CI: 61.8 - 65.6) seeking dental care within the past year (Figure 3 below).

Adults who have visited a dentist or dental clinic in the past year based on education level

The education categories were divided into five types: less than high school (H.S.), high school or equivalent, such as general educational development (G.E.D.), some post-H.S. education, and college graduate or professional degree. These categories were employed to explore variations in dental care utilization among individuals with varying levels of educational attainment. The results revealed variations in the proportion of adults who visited a dentist or dental clinic in the past year based on different education levels (Table 4 below).

**Table 4:** Classification by education level of individuals who have visited a dentist or dental clinic in the past year CI: Confidence interval, (-): Intentionally left blank, n: number

-	Less than H.S.	H.S. or G.E.D.	Some post H.S.	College graduate
2020				
Percent (%)	43.8	59.6	65.8	77.3
CI	42.4 - 45.2	58.9 - 60.3	65.1 - 66.5	76.8 - 77.8
n	10255	60834	73053	121800
2018				
Percent (%)	46.1	60.8	68	80
CI	45.0 - 47.2	60.2 - 61.4	67.5 - 68.5	79.6 - 80.4
n	13186	68834	80382	132720
2016				
Percent (%)	44.4	60.1	68	79.6
CI	43.4 - 45.4	59.6 - 60.6	67.5 - 68.5	79.2 - 80.0
n	14395	77843	88782	141296
2014				
Percent (%)	43.3	58.9	66.8	79.3
CI	42.3 - 44.3	58.4 - 59.4	66.3 - 67.3	78.9 - 79.7
n	13622	74594	83326	133663
2012				
Percent (%)	42.6	60.8	68	80.4
CI	41.6 - 43.6	60.3 - 61.3	67.5 - 68.5	80.0 - 80.8
n	15741	80621	85923	131540

**Figure 4:**
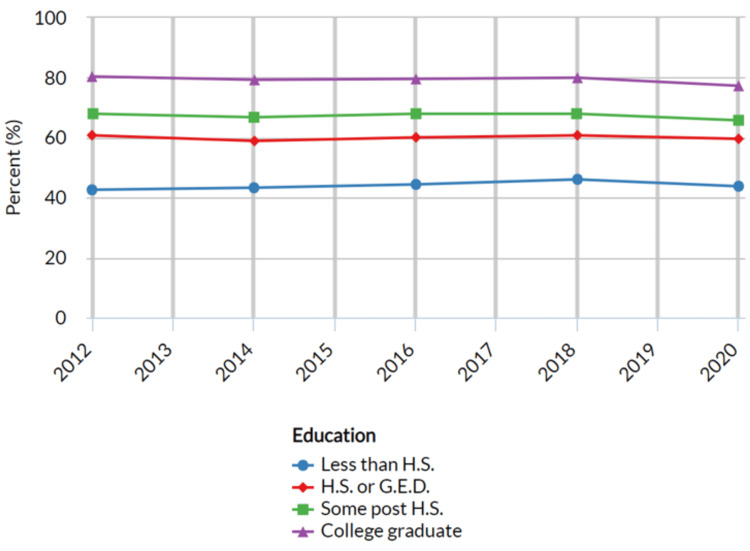
Adults who have visited a dentist or dental clinic in the past year based on education United States- All available years, Adults aged 18years+ who have visited a dentist or dental clinic in the past year, Breakdown – Education, Response-Yes, H.S-High school, G.E.D-Graduate education

Among the education groups considered, the highest proportion of adults seeking dental care in the past year was observed among college graduates or those with professional degrees, reporting a proportion of 77.3% (n = 121,800; 95% CI: 76.8 - 77.8) in 2020. As education levels decreased, the proportion of dental care seekers also decreased. Adults with some post-H.S. education exhibited a proportion of 65.8% (n = 73,053; 95% CI: 65.1 - 66.5). Similarly, among those with a high school diploma or equivalent (G.E.D.), the proportion was 59.6% (n = 60,834; 95% CI: 58.9 - 60.3). For individuals with less than a high school education, the proportion seeking dental care was 43.8% (n = 10,255; 95% CI: 42.4 - 45.2) (Figure 4 below).

Adults who have visited a dentist or dental clinic in the past year based on age

Age categories (in years) were defined in the analysis as follows: 18-24, 25-34, 35-44, 45-54, 55-64, and 65+. These age groups were utilized to explore variations in dental care utilization across the lifespan. The results revealed insignificant variations in the proportion of adults who visited a dentist or dental clinic in the past year across different age categories (Table 5 below).

**Table 5:** Classification by age of individuals who have visited a dentist or dental clinic in the past year CI: Confidence interval, (-): Intentionally left blank, n: number

Age (Years)	18–24	25–34	35–44	45–54	55–64	65+
2020	-	-	-	-	-	-
Percent (%)	66.6	59.2	63.3	65.9	66.5	67.1
CI	65.4 - 67.8	58.3 - 60.1	62.4 - 64.2	65.0 - 66.8	65.6 - 67.4	66.4 - 67.8
n	16923	26249	33700	41633	52528	96012
2018	-	-	-	-	-	-
Percent (%)	69.3	60.8	65.5	68.1	68.3	67.8
CI	68.3 - 70.3	60.0 - 61.6	64.7 - 66.3	67.4 - 68.8	67.6 - 69.0	67.2 - 68.4
n	17598	28517	35074	46141	62107	106610
2016	-	-	-	-	-	-
Percent (%)	68.6	60.2	65.2	66.1	68	66.9
CI	67.7 - 69.5	59.4 - 61.0	64.4 - 66.0	65.4 - 66.8	67.4 - 68.6	66.4 - 67.4
n	17500	29010	36304	51443	73583	115459
2014	-	-	-	-	-	-
Percent (%)	67.8	57.3	63.6	65.8	67.1	65.7
CI	66.8 - 68.8	56.5 - 58.1	62.8 - 64.4	65.1 - 66.5	66.5 - 67.7	65.2 - 66.2
n	15468	25704	36084	52040	72777	105435
2012	-	-	-	-	-	-
Percent (%)	67.2	59.7	64.9	66.5	68.8	66
CI	66.2 - 68.2	58.9 - 60.5	64.1 - 65.7	65.8 - 67.2	68.2 - 69.4	65.5 - 66.5
n	16013	29504	40591	56677	73392	98815

**Figure 5:**
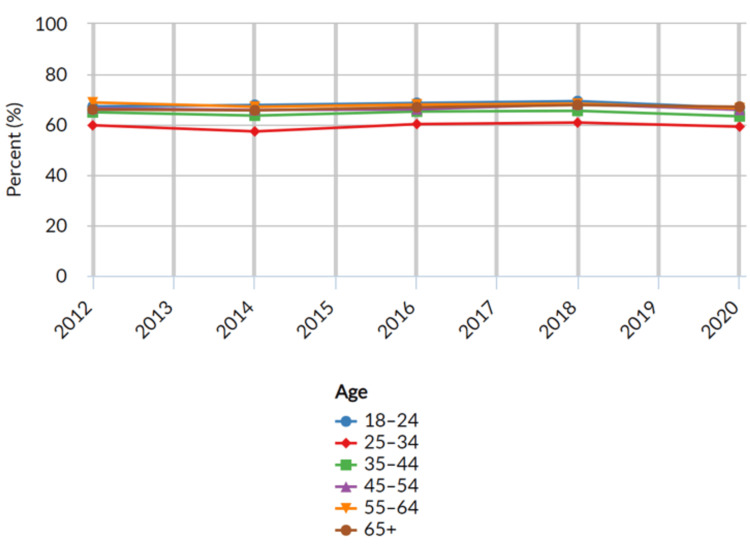
Adults who have visited a dentist or dental clinic in the past year based on age United States- All available years, Adults aged 18 years+ who have visited a dentist or dental clinic in the past year, Breakdown – Age, Response-Yes

Among the age groups considered, the highest proportion of dental care seekers was observed among adults aged 65 years and above, reporting a proportion of 67.1% (n = 96,012; 95% CI: 66.4 - 67.8) in 2020. However, as age decreased, the proportion of dental care seekers generally followed similar trends within the same range. Adults aged 55-64 reported a proportion of 66.5% (n = 52,528; 95% CI: 65.6 - 67.4), followed by those aged 18-24 with a proportion of 66.6% (n = 16,923; 95% CI: 65.4 - 67.8). Among adults aged 45-54, the proportion seeking dental care was 65.9% (n = 41,633; 95% CI: 65.0 - 66.8), while for the 35-44 age group, the proportion was 63.3% (n = 33,700; 95% CI: 62.4 - 64.4). Adults aged 25-34 exhibited the lowest proportion of dental care seekers, with 59.2% (n = 26,249; 95% CI: 58.3 - 60.1) visiting a dentist or dental clinic in the past year (Figure 5 below).

## Discussion

The investigation into dental care utilization patterns among adults aged 18 and above who have visited a dentist or dental clinic in the past year, with a focus on gender, education, age, income, and race, offers a comprehensive understanding of the multifaceted dynamics influencing oral health practices. This discussion delves into the implications of the findings, explores potential contributing factors, and contextualizes the broader implications for oral health promotion and equitable access to care.

Our analysis revealed that 64% of adults aged 18 and above reported visiting a dentist or dental clinic in the year 2020. This proportion reflects a significant portion of the population that prioritized their oral health and engaged in preventive care measures. The figure underscores the recognition of the importance of regular dental visits in maintaining oral health and preventing potential dental issues from escalating. This observation is consistent with the understanding that routine dental care is a cornerstone of preventive healthcare, contributing to the overall well-being of individuals [12-13]. The study outlined trends in dental care utilization before (in 2018) and during the COVID-19 pandemic (in 2020). While 66.5% of adults aged 18 and above had reported visiting a dentist or dental clinic in 2018 before the pandemic, this proportion decreased to 64.8% in 2020 during the pandemic period. These findings aligned with the retrospective, observational study conducted by Kranz et al., which reported a significant decline in weekly visits to US dental offices during the initial phases of the COVID-19 pandemic [14].

The study's findings revealed gender disparities in dental care utilization, with implications that went beyond oral health. While both males and females engaged in dental care, the proportions were higher in females, reflecting potential sociocultural factors influencing healthcare behaviors [13,15]. A cross-sectional study conducted by Su et al. also reported that females (53%) were more proactive in visiting dental clinics than their male counterparts (47%) [15]. These observed gender-related disparities in dental care utilization aligned with broader healthcare-seeking trends, wherein females often demonstrated higher rates of engagement with healthcare services, including dental care [15]. Factors such as greater health consciousness, willingness to address health concerns, and societal norms may have contributed to the observed differences in dental care-seeking behavior between genders. However, it's important to note that gender is a complex construct influenced by various social and cultural factors that may interact with oral health behaviors [16]. The analysis highlighted a significant association between education level and dental care utilization. Individuals with higher education levels tended to have a greater likelihood of visiting a dentist or dental clinic in the past year. The observed education-related disparities in dental care utilization were consistent with the notion that education played a crucial role in health-related behaviors, including healthcare-seeking [15,17]. Higher levels of education were often associated with better health knowledge, increased health literacy, and greater awareness of the importance of preventive care, such as regular dental visits [17]. A study conducted by Baskaradoss JK reported that individuals with low education had limited oral health literacy levels and resulted in delayed or missed dental visits. Conversely, individuals with lower education levels may have faced barriers such as limited health literacy and financial constraints that impacted their ability to seek timely dental care [18].

Income disparities in dental care utilization underscored the interplay between socioeconomic factors and access to healthcare services. Individuals with higher incomes were more likely to seek dental care, possibly due to greater financial resources and health insurance coverage. Conversely, individuals with lower incomes may have faced barriers to accessing dental care, including financial constraints and limited availability of affordable services. The observed income-related disparities in dental care utilization were consistent with previous research highlighting the influence of socioeconomic factors on healthcare access, including oral health services [15,19]. Limited financial resources, lack of dental insurance coverage, and other economic barriers may have contributed to the observed variations in dental care-seeking behavior among different income groups [19].

The analysis of dental care utilization based on race highlighted disparities that may have stemmed from cultural, systemic, and economic factors. Differences in oral health practices among racial groups could have been influenced by cultural beliefs, historical disparities in healthcare access, and structural inequalities, including low income or lack of private health insurance, distance from dental clinics, and racism. Aligned with our findings, a study undertaken by Sabbah et al. aimed at investigating racial discrimination in dental visit patterns among American adults similarly revealed that the highest proportion was among White individuals (72.5%), followed by Black individuals (56.7%), and Hispanic individuals (57.0%) [20]. The findings aligned with existing literature highlighting racial disparities in oral health outcomes and healthcare access. Factors such as cultural norms, socioeconomic status, language barriers, and systemic inequalities may have contributed to these variations in dental care-seeking behavior among different racial groups [15,18-21]. The study's investigation into age-related disparities in dental care utilization highlighted how health priorities changed across various life stages. However, it's important to note that the proportion of dental visits did not exhibit significant variations. The observed age-related disparities in dental care utilization may have reflected shifts in priorities and awareness of oral health needs as individuals progressed through different life stages. Older adults tended to have more regular dental check-ups as they recognized the importance of maintaining oral health in their later years [15]. 

This study examining dental care utilization among adults aged 18 and above who had visited a dentist or dental clinic in the past year benefited from several strengths and acknowledged certain limitations. The utilization of data from the CDC Oral Health dataset provided a solid foundation, offering a nationally representative sample that enhanced the generalizability of findings. The inclusion of a wide range of demographic variables, such as age, gender, income, education, and race, allowed for an in-depth exploration of factors influencing dental care-seeking behavior. Furthermore, the study's focus on dental care practices among adults held significant relevance for public health, guiding interventions and policies to improve oral health outcomes. The other notable strength of this study entails the observation that the use of CDC Oral Health data offers detailed qualitative data, which enables a nuanced evaluation of the rationales underlying the behavior of individuals in the study populations. The data from the CDC, alongside the findings of the study, can aid in shaping public health campaigns while also informing the dental practitioners’ approaches to educating individuals aged 18 years and above on proper dental hygiene.

However, it's important to recognize the limitations inherent in this analysis. Relying on self-reported data for dental care utilization introduced potential recall and social desirability biases. While the study revealed associations, causality could not be inferred due to unmeasured confounding factors. Moreover, the analysis did not encompass all potential variables influencing dental care decisions, such as dental insurance coverage or cultural beliefs. The findings should be interpreted with consideration of the specific year's context, as dental care utilization trends could have evolved due to changing healthcare policies and economic conditions. Lastly, the data utilized was collected in 2019 and 2020, which was an increasingly dynamic period in which opinions and thoughts regarding COVID-19 might have an immense impact on dental healthcare-seeking behaviors. This might have effects on the amount and quality of data collected. Despite these limitations, the study contributed to our understanding of oral health practices and informed future research and interventions aimed at enhancing equitable dental care access and utilization.

## Conclusions

The study's main conclusion underscores the importance of regular dental visits for maintaining optimal oral health. The reported proportion of adults who visited a dentist or dental clinic in the past year reflects a collective recognition of the value of preventive dental care and its role in preventing potential oral health problems. Disparities in dental care use based on age, gender, income, education, and race highlight the intricate interplay of factors influencing oral health behaviors. These findings offer valuable insights for public health policymakers, dental practitioners, and researchers. Targeted interventions addressing barriers faced by specific subgroups and promoting oral health education could help reduce these disparities and enhance fair access to dental care.
